# Asciminib in Patients With CML-CP Previously Treated With ≥ 2 Tyrosine Kinase Inhibitors: 96-Week Results From the Japanese Subgroup Analysis of the ASCEMBL Study

**DOI:** 10.1007/s12185-024-03805-0

**Published:** 2024-06-18

**Authors:** Yosuke Minami, Noriko Doki, Hiroshi Matsuoka, Takafumi Yokota, Akihiro Tomita, Naoto Takahashi, Kohmei Kubo, Tatsunori Goto, Keita Kirito, Akio Maki, Makoto Aoki, Meryem Ktiouet Dawson, Itaru Matsumura

**Affiliations:** 1 Department of Hematology, National Cancer Center Hospital East, Chiba, Kashiwa 277-8577 Japan; 2 Tokyo Metropolitan Cancer and Infectious Diseases Center, Komagome Hospital, Tokyo, Japan; 3 Kobe University Hospital, Kobe, Japan; 4 Osaka University Hospital, Osaka, Japan; 5 Osaka International Cancer Institute, Osaka, Japan; 6 Fujita Health University School of Medicine, Toyoake, Japan; 7 Akita University Hospital, Akita, Japan; 8 Aomori Prefectural Central Hospital, Aomori, Japan; 9 Japanese Red Cross Aichi Medical Center Nagoya Daiichi Hospital, Nagoya, Japan; 10 University of Yamanashi Hospital, Yamanashi, Japan; 11 Novartis Pharma K.K., Tokyo, Japan; 12 Novartis Pharma AG, Basel, Switzerland; 13 Kindai University Hospital, Osaka, Japan

**Keywords:** Asciminib, BCR, ABL1 inhibitor, Chronic myeloid leukemia, Major molecular response, STAMP, Tyrosine kinase inhibitors

## Abstract

Asciminib is a first-in-class BCR::ABL1 inhibitor that Specifically Targets the ABL1 Myristoyl Pocket (STAMP). It is approved worldwide and in Japan for chronic myeloid leukemia in chronic phase (CML-CP) with resistance or intolerance to previous tyrosine kinase inhibitor (TKI) therapy. In the Phase 3 ASCEMBL study, patients with CML-CP who received ≥ 2 prior ATP-competitive TKIs were randomized (2:1) to asciminib 40 mg twice-daily or bosutinib 500 mg once-daily. Here, we report the 96-week results of the subgroup analysis of Japanese patients (asciminib, *n* = 13; bosutinib, *n* = 3) in the ASCEMBL study. The MMR rate at Week 96 was 46.2% in asciminib-treated patients, increasing from Weeks 24 and 48. Patients who achieved MMR at Week 24 remained in MMR up to the Week 96 cutoff. While a high proportion of patients treated with asciminib remained on treatment at cutoff, none randomized to bosutinib were on treatment at Week 96. Despite the longer duration of exposure to asciminib, its safety and tolerability continued to be favorable with no new or worsening safety findings. Overall, the efficacy and safety outcomes in the Japanese subgroup were comparable with the ASCEMBL global study population, which supports the use of asciminib in Japanese patients with previously treated CML-CP.

## Introduction

The introduction of tyrosine kinase inhibitors (TKIs) has changed the treatment landscape of chronic myeloid leukemia (CML), leading to a substantial improvement in the survival of patients with a life expectancy comparable to that of the average population [1]. In Japan, the latest treatment guidelines recommend the use of imatinib, nilotinib, dasatinib, and bosutinib for the treatment of newly diagnosed patients with CML in chronic phase (CML-CP) with consideration to comorbidities and other patient characteristics. For patients with resistance or intolerance to first-line TKIs, Japanese treatment guidelines recommend switching to a different second-generation TKIs (nilotinib, dasatinib, or bosutinib) and third-generation ponatinib or asciminib for patients previously treated with ≥ 2 TKIs﻿ [2].

The main goal of treatment for CML-CP is to prevent progression to blast phase and to achieve durable deep molecular responses (DMR). The use of second- and third-generation TKIs has increased the likelihood of achieving DMR and therefore, the treatment goals have shifted over time from prolonging survival, preventing disease progression, and reducing treatment-related toxicities to achieving long-term treatment-free remission (TFR) [3–5]. However, some patients may not have an optimal response to treatment, or they may lose response, or experience intolerance and fail to achieve DMR with the currently approved adenosine triphosphate (ATP)-binding TKIs.

Asciminib is a first-in-class BCR::ABL1 inhibitor that acts by Specifically Targeting the ABL1 Myristoyl Pocket (STAMP), a mechanism of action distinct from that of other TKIs. Asciminib inhibits the kinase activity of BCR::ABL1 by allosterically binding to the myristoyl pocket of ABL1; as such, asciminib is expected to maintain activity against mutations that involve the ATP-binding﻿ site of BCR::ABL1 [6, 7]. On the basis of results from the Phase 1 dose-finding study (NCT02081378) in heavily pre-treated patients and the pivotal Phase 3 ASCEMBL study (NCT03106779), asciminib was approved for the first time in the world by the United States Food and Drug Administration in October 2021 for the treatment of patients with Philadelphia chromosome-positive﻿ (Ph +) CML-CP previously treated with ≥ 2 TKIs and for adults with Ph + CML-CP harboring the T315I mutation [8]. Asciminib is also approved by the Ministry of Health, Labour, and Welfare in Japan for the treatment of CML patients with resistance or intolerance to previous TKI therapy in March 2022, followed by the approval by the European Medicines Agency for the treatment of adult patients with Ph + CML-CP previously treated with ≥ 2 TKIs in August 2022.

We have previously reported the results from the Week 24 primary analysis from the pivotal ASCEMBL study, in which asciminib demonstrated statistically significant and superior efficacy and better safety and tolerability than bosutinib in patients with CML-CP treated with ≥ 2 prior TKIs. Briefly, asciminib monotherapy was more effective than bosutinib monotherapy in terms of the proportion of patients achieving major molecular response (MMR, *BCR::ABL1*^IS^ ≤ 0.1%) at Week 24 (25.5% vs. 13.2%, respectively; between-group difference 12.2% [95% CI 2.19–22.30, *p* = 0.029] after adjusting for major cytogenetic response). The proportions of patients who reported grade ≥ 3 adverse events (AEs; 50.6% vs. 60.5%, respectively) and those who discontinued treatment due to AEs (5.8% vs. 21.1%, respectively) were lower with asciminib compared to bosutinib [9]. The differences in the MMR rate between the two treatment arms were maintained at Week 48 with no new safety findings or worsening of AEs compared to Week 24 [10]. The Week 24 subgroup analysis of the Japanese patients enrolled in the ASCEMBL study showed that the efficacy and safety results observed in the overall population were comparable to those reported for Japanese patients at this time point [11].

Recently, the 96-week analysis of the ASCEMBL study comparing the long-term benefits and risks of asciminib with those of bosutinib in patients with CML-CP treated with ≥ 2 prior TKIs was reported. The results from long-term follow-up data (median follow-up, 2.3 years) showed that the benefits of asciminib in terms of MMR as early as Week 12 were maintained up to Week 96. The key secondary endpoint of MMR rate at Week 96 was 37.6% with asciminib and 15.8% with bosutinib; the MMR rate difference (adjusted by baseline major cytogenetic response [MCyR]) was 21.74% (95% CI, 10.53–32.95; two-sided *p* = 0.001). The cumulative incidence of MMR by Week 96 was higher with asciminib compared with bosutinib. Despite the longer duration of exposure, the overall safety profile of asciminib at Week 96 remained consistent with that observed during Week 24 [12].

Here we report the longer-term efficacy and safety results of the ASCEMBL study after a median follow-up of 96 weeks in the Japanese subgroup of enrolled patients (72 weeks after primary analysis), to determine whether the results observed in Japanese patients are comparable with the global population.

## Methods

### Study design and patients

The study design and patient eligibility criteria for the ASCEMBL (NCT03106779) study have been previously described in detail [9]. Briefly, ASCEMBL is a randomized, open-label, active-controlled, multicenter, Phase 3 study investigating the efficacy and safety of asciminib vs. bosutinib in the treatment of patients with CML-CP who received ≥ 2 prior ATP-competitive TKIs. Patients were randomized (2:1) to receive either asciminib 40 mg twice daily or bosutinib 500 mg once daily. The randomization was stratified by the presence or absence of major cytogenetic response (MCyR) at baseline. Adult patients with CML-CP previously treated with  ≥ 2 TKIs with an Eastern Cooperative Oncology Group (ECOG) performance status of ≤ 2 and experiencing treatment failure (as per 2013 European LeukemiaNet [ELN] recommendations) or intolerance to their most recent TKI were included. Patients with intolerance to their most recent TKI were required to have *BCR::ABL1*^IS^ > 0.1% at screening. Those harboring T315I or V299L mutations were excluded. A protocol amendment on December 14, 2018 allowed patients experiencing treatment failure with bosutinib treatment (as per 2013 ELN recommendations) to switch to asciminib treatment within 96 weeks after the last patient was randomized in the study. Data collected for patients receiving asciminib after switching from bosutinib are not included in the present analysis. This manuscript presents the results of key secondary endpoint analyses based on the subgroup of patients treated in Japan for 96 weeks within the ASCEMBL study.

## Treatments

Asciminib and bosutinib were taken orally as tablets. Patients received study treatment for up to 96 weeks after the last patient received the first dose or up to 48 weeks after the last patient in the bosutinib arm had switched to asciminib treatment, whichever was longer. Concomitant medications/therapies deemed necessary for the supportive care of the patients were permitted. Patients meeting lack of efficacy criteria were to permanently discontinue study treatment. Patients could voluntarily withdraw from the study for any reason at any time and those who discontinued or switched treatment during the treatment period entered the survival follow-up phase, except for reasons of death, loss to follow-up, or consent withdrawal.

## Endpoints and assessments

The details of the primary efficacy and safety assessments in the Japanese subgroup of patients have been reported previously [11]. The key secondary objective was to assess the efficacy of asciminib vs. bosutinib at 96 weeks in patients with CML-CP treated with ≥ 2 prior TKIs. The key secondary endpoint included the MMR rate at 96 weeks in patients treated with asciminib vs. bosutinib. In addition, the other secondary endpoints included MMR at all scheduled timepoints (including Weeks 24 and 96), time to MMR and duration of MMR, *BCR::ABL1*^*IS*^ ≤ 1% at 24, 48, and 96 weeks, and MR^4^ (*BCR::ABL1*^*IS*^ ≤ 0.01%) and MR^4.5^ (*BCR::ABL1*^*IS*^ ≤ 0.0032%) at 24, 48, and 96 weeks. For patients to be counted as being in MMR at Week 96, they must have been on study treatment with *BCR::ABL*^IS^ ≤ 0.1% at 96 weeks and not met treatment failure criteria prior to 96 weeks. In addition, the results of a self-reported patient-reported outcome (PRO) questionnaire, the MD Anderson Symptom Inventory–chronic myeloid leukemia (MDASI-CML) which is a validated 26-item, multi-symptom PRO questionnaire for clinical and research use, are reported. The safety and tolerability profile of asciminib vs. bosutinib at Week 96 is also reported.

## Statistical analysis

The MMR rate at 96 weeks was calculated using the full analysis set (FAS). All Japanese patients who were randomized to study treatment in the ASCEMBL study were included in the FAS and are part of this subgroup analysis. The Cochran–Mantel–Haenszel chi-square test stratified by baseline MCyR status (MCyR vs. no MCyR) was used to compare the MMR rates between the treatment groups for the global trial whole population. Due to the limited number of patients in the Japanese subgroup, no statistical testing was performed between the treatment arms. The MMR rate and the associated two-sided 95% CI, calculated based on the Clopper-Pearson method, are presented.

Patients without polymerase chain reaction (PCR) assessment at Week 96 were considered as ﻿non-responders, unless both the 84-week and 108-week PCR assessments indicated that the patient was in MMR. This subgroup analysis included data collected up to the data cutoff date of October 6, 2021, when all the randomized patients had completed their Week 96 visit or had discontinued earlier. All the safety analyses were based on the safety set population, which included all Japanese patients who received at least one dose of study drug.

## Ethics

The protocol was approved by the independent ethics committee or institutional review board at each site, and the study was conducted in accordance with the principles of the Declaration of Helsinki. All patients provided written informed consent. An independent data monitoring committee reviewed safety data approximately every 6 months.

## Results

### Baseline characteristics and patients’ status

Overall, 16 patients across 10 Japanese sites were randomized in the ASCEMBL study to receive asciminib (*n* = 13) or bosutinib (*n* = 3).

The baseline characteristics of the subgroup of patients treated in Japan have been described in detail previously [11]. The comparison of baseline characteristics is limited due to the low number of Japanese patients randomized to the bosutinib group. The median age of patients treated with asciminib was 56.0 years and in the bosutinib group it was 40.0 years. At baseline, 7 patients (53.8%) had *BCR::ABL1*^*IS*^ > 1 to ≤ 10%, and 3 patients (23.1%) each had *BCR::ABL1*^*IS*^ > 0.1% to ≤ 1 and *BCR::ABL1*^*IS*^ > 10% in the asciminib group. In the bosutinib group, all 3 patients had *BCR::ABL1*^*IS*^ > 10% at baseline. Among the complete cytogenetic response (CCyR) analysis set (*n* = 8), 4 patients were in MCyR at baseline. The most common reasons for discontinuation of the last prior TKI were lack of efficacy in 7 patients and lack of tolerability in 6 patients in the asciminib group. In the bosutinib group, the reason for discontinuation was lack of efficacy in 2 patients and lack of tolerability in 1 patient.

At the time of data cutoff, treatment was ongoing for 10 patients (76.9%) in the asciminib group, while none were on treatment in the bosutinib group. Three patients discontinued treatment in the asciminib group between Weeks 24 and 48: two patients due to AEs and one patient due to lack of efficacy (Table 1). In the bosutinib group, all three patients discontinued treatment by Week 48, of which two patients discontinued due to AEs and one due to lack of efficacy.

**Table 1:** Patient’s status until Week 96

Patients, *n* (%)	Japanese subgroup	
	Asciminib 40 mg BID (*n* = 13)	Bosutinib 500 mg QD (*n* = 3)
Treated	13 (100)	3 (100)
Treatment ongoing*	10 (76.9)	0
Discontinued from treatment	3 (23.1)	3 (100)
< Week 24	0	2 (66.7)
≥ Week 24 and < Week 48	3 (23.1)	1 (33.3)
≥ Week 48 and < Week 96	0	0
≥ Week 96	0	0
Reason for discontinuation		
Lack of efficacy	1 (7.7)	1 (33.3)
Adverse event	2 (15.4)	2 (66.7)
Switched to receive asciminib	NA	1 (33.3)

^*^Ongoing at the time of data cutoff: October 6, 2021

*BID* twice daily; *QD* once daily; *NA*, not applicable

## Efficacy

### MMR rate at Week 96

**Fig. 1:**
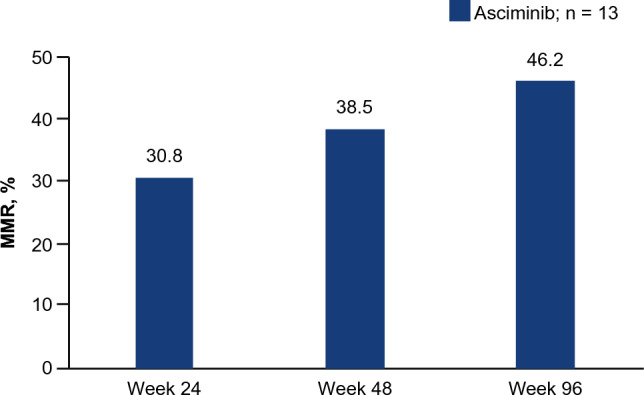
MMR rate over 96 weeks of treatment with asciminib in Japanese patients. *MMR*, major molecular response

Only a small number of Japanese patients were randomized to the bosutinib arm and since none of the patients in the bosutinib arm remained on treatment by the 96-week cutoff, here we describe the efficacy results of the asciminib group in the Japanese subgroup. The MMR rate at Week 96 was 46.2% (95% CI: 19.22–74.87) in the asciminib-treated group (the key secondary endpoint of the ASCEMBL study), showing an increase from Week 24 and Week 48. MMR rates in the Japanese subgroup are shown in Fig. 1. Among the 6 Japanese patients who were in MMR at Week 96, the median time to first MMR was 12.1 (range: 4‒36) weeks. All patients who had achieved MMR with asciminib at Week 24 remained in MMR up to the Week 96 cutoff.

## ***BCR::ABL1***^***IS***^ ≤ 1% at Week 96

In the Japan subgroup, the proportion of patients with *BCR::ABL1*^*IS*^ ≤ 1% at Week 96 in the asciminib arm was 61.5% (Table 2). The proportion of patients with *BCR::ABL1*^*IS*^ ≤ 1% in patients treated with asciminib remained constant over 24, 48, and 96 weeks. None of the patients on bosutinib had *BCR::ABL1*^*IS*^ ≤ 1% at Week 96.

**Table 2:** Efficacy in Japanese patients randomized to asciminib

Characteristic	Japanese subgroup		
	**Asciminib**		
*n* (%)	**Week 24**	**Week 48**	**Week 96**
Molecular response (*N* = 13)			
* BCR::ABL1*^*IS*^ ≤ 1%	8 (61.5)	8 (61.5)	8 (61.5)
MMR *(BCR::ABL1*^*IS*^ ≤ 0.1%)	4 (30.8)	5 (38.5)	6 (46.2)
MR^4^ *(BCR::ABL1*^*IS*^ ≤ 0.01%)	1 (7.7)	1 (7.7)	1 (7.7)
MR^4.5^ (*BCR::ABL1*^*IS*^ ≤ 0.0032%)	1 (7.7)	1 (7.7)	1 (7.7)
Cytogenetic response (*N* = 8)			
CCyR	4 (50.0)	3 (37.5)	3 (37.5)

*CCyR* complete cytogenetic response; *IS* international scale; *MMR* major molecular response; *MR* molecular response

## MR^4^ and MR^4.5^ at Week 96

Only one patient (7.7%) treated with asciminib was in MR^4.5^ (*BCR::ABL1*^IS^ ≤ 0.0032%) at Week 24; this patient maintained this response at Week 96 (Table 2). The same patient was also considered as having achieved MR.^4^

## Cytogenetic response

The CCyR rate at Week 24 was 50.0% (95% CI: 15.70–84.30) with asciminib (*N* = 8), which later decreased to 37.5% (95% CI: 8.52–75.51) at Week 48 and remained at this level until the 96-week﻿ cutoff date (Table 2). This decrease in the CCyR response rate is due to one patient who missed the bone marrow assessment owing to study discontinuation before Week 48.

## Mutations

At baseline, no patients harbored any *BCR::ABL1* mutation. No patients harbored post-baseline newly emerged *BCR::ABL1* gene mutations at Week 96.

## Patient-reported outcomes

At baseline, the MDASI-CML total severity score (mean [SD]) was 1.4 (1.38) in Japanese patients randomized to the asciminib treatment arms. The mean total severity scores improved over 96 weeks of asciminib treatment, and these improvements were seen as early as Week 4. The change from baseline of total severity scores (mean [SD]) throughout asciminib treatment were − 0.7 (1.64; *n* = 11) at Week 24, − 0.5 (2.77; *n* = 8) at Week 48, and − 0.7 (1.91; *n* = 10) at Week 96. The change from baseline was calculated for patients who had the assessment of both baseline and respective post-baseline.

## Safety

In the asciminib-treated group, the median duration of exposure by the cutoff date was 116.14 weeks (range: 20.1–172.9), whereas in the bosutinib group, it was 3.14 weeks (range: 1.3–29.0). In patients who received asciminib, the median average daily dose was 79.9 mg (range: 39–80), and in those treated with bosutinib, it was 500.0 mg (range: 500–500).

In the Japanese subgroup of patients, 11 patients (84.6%) in the asciminib group and 3 patients (100.0%) in the bosutinib-treated group experienced at least one AE of any grade during the study observation period. The most common AEs (occurring in ≥ 20% of all patients and in ≥ 2 patients in any treatment arm of the Japanese subgroup) observed with asciminib were neutropenia (38.5%), nasopharyngitis (30.8%), and thrombocytopenia (23.1%), whereas the most common AEs reported with bosutinib were diarrhea (100%), neutropenia (66.7%), and constipation (66.7%) (Table 3).

**Table 3:** Adverse events

Category, *n* (%)	Japanese subgroup			
	Asciminib 40 mg BID (*n* = 13)		Bosutinib 500 mg QD (*n* = 3)	
	All grades	Grade ≥ 3	All grades	Grade ≥ 3
Number of patients with at least one event	11 (84.6)	7 (53.8)	3 (100)	2 (66.7)
Most frequent AEs*				
Thrombocytopenia^†^	3 (23.1)	2 (15.4)	1 (33.3)	0
Neutropenia^‡^	5 (38.5)	4 (30.8)	2 (66.7)	1 (33.3)
Diarrhea	1 (7.7)	0	3 (100.0)	0
Constipation	0	0	2 (66.7)	0
Nasopharyngitis	4 (30.8)	0	0	0

^*^Only the most frequent AEs (occurring in ≥ 20% of patients in any treatment arm in all patients and AEs occurring in ≥ 2 patients in any treatment arm) in the Japanese patient subgroup are shown. Numbers represent counts of patients. A patient with multiple severity grades for an AE is only counted under the maximum grade; MedDRA version 24.1, CTCAE version 4.03

^†^Grouped term including AEs reported by investigator as thrombocytopenia and platelet count decreased

^‡^Grouped term including AEs reported by investigator as neutropenia and neutrophil count decreased

*AE* adverse event; *BID* twice daily; *CTCAE* Common Terminology Criteria for Adverse Events; *MedDRA* Medical Dictionary for Regulatory Activities; *QD* once daily

**Fig. 2:**
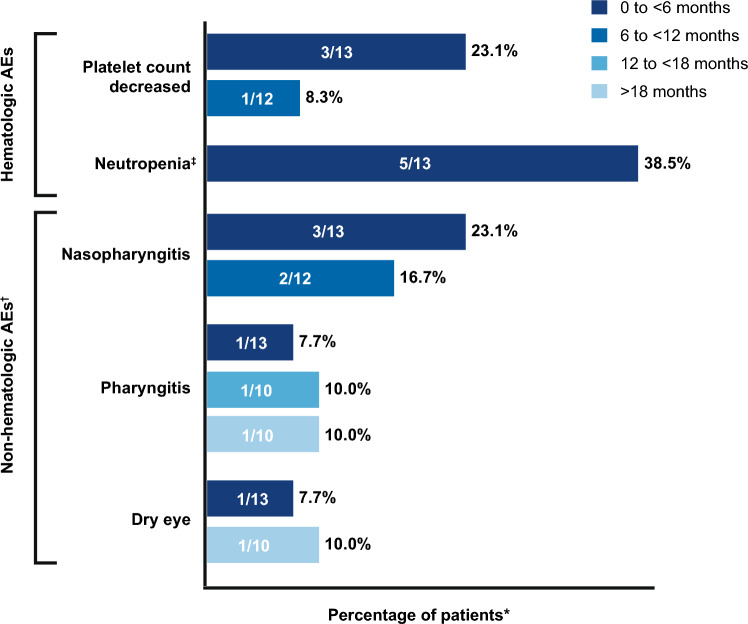
All-grade AEs by time period with asciminib*. *A patient with multiple occurrences of an adverse event is counted only once in that time period. Adverse events which occurred repeatedly over the different time periods were counted at each period, respectively. †Adverse events occurring in ≥ 2 patients across time-points are shown. ‡Includes neutropenia and neutrophil count decreased

In patients treated with asciminib, most AEs (69.2%) occurred initially within the first 6 months of treatment initiation, and these AEs rarely persisted beyond the time period of their initial presentation. Neutropenia (included neutrophil count decreased) and decreased platelet count were the most common AEs which occurred within the first 6 months of treatment with asciminib (Fig. 2). No deaths were reported during the 96-week treatment period in the Japanese patient subgroup.

## Discussion

The efficacy and safety results in the overall ASCEMBL study population over 96 weeks of treatment showed increasingly superior efficacy for asciminib compared with bosutinib in patients with CML-CP treated with ≥ 2 prior TKIs [12]. This subgroup analysis evaluated the longer-term efficacy and safety of asciminib vs. bosutinib in Japanese patients with CML-CP treated in the ASCEMBL study for 96 weeks.

Among Japanese patients treated with asciminib, the MMR rate at Week 96 was higher compared to that observed at Week 24 (46.2% vs. 30.8%, respectively), with a consistent trend showing an increase in MMR rate over the 96 weeks of treatment. The asciminib MMR rate at Week 96 was higher in the Japanese subgroup of patients compared with the overall ASCEMBL study population (37.6%) (Table 4). When comparing the results of this subgroup analysis with the overall population, it should be noted that the Japanese subgroup included a limited number of patients, which may affect the results. As reported previously, none of the patients receiving bosutinib (*n* = 3) achieved MMR, CCyR, or *BCR::ABL1*^*IS*^ ≤ 1% at Week 24 in the Japanese subgroup. By Week 48, all patients assigned to the bosutinib group had discontinued treatment due to treatment failure or AEs. As there were no patients in the bosutinib group, efficacy comparisons for asciminib vs. bosutinib cannot be drawn from this Japanese subgroup analysis. MMR is an important treatment goal in CML: achieving it could lower the risk of disease progression and result in attaining DMR and ultimately improved survival [5, 13]. However, in patients who have failed multiple lines of TKI therapy, treatment with a different second-generation TKI may not lead to deeper or durable responses [14]. In the ASCEMBL overall population and in the Japanese patient subgroup, however, a substantial number of patients who had failed treatment with prior TKIs achieved MMR with asciminib. The Japanese patients who achieved MMR with asciminib at Week 24 continued to be in MMR at Week 96. The number of patients who achieved *BCR::ABL1*^IS^ ≤ 1% with asciminib was consistent across 24, 48, and 96 weeks. These results suggest that the clinical benefits observed with asciminib are durable and may even improve over time.

**Table 4:** Week 96 efficacy and safety in Japanese patients and in all patients randomized to asciminib in the ASCEMBL study

*n* (%)	ASCEMBL	
	Japanese patients randomized to asciminib (*n* = 13)	All patients randomized to asciminib (*n* = 157)
Molecular response at Week 96		
* BCR::ABL1*^IS^ ≤ 1%	8 (61.5)	64 (45.1)
MMR (*BCR::ABL1*^IS^ ≤ 0.1%)	6 (46.2)	59 (37.6)
MR^4^ (*BCR::ABL1*^IS^ ≤ 0.01%)	1 (7.7)	27 (17.2)
MR^4.5^ (*BCR::ABL1*^IS^ ≤ 0.0032%)	1 (7.7)	17 (10.8)
Cytogenetic response at Week 96		
CCyR	3 (37.5)	41 (39.8)
Safety		
AEs	11 (84.6)	142 (91.0)
Grade ≥ 3 AEs	7 (53.8)	88 (56.4)
Treatment-related AEs	7 (53.8)	104 (66.7)
SAEs	1 (7.7)	28 (17.9)
Fatal SAEs	0	2 (1.3)
AEs leading to discontinuation	2 (15.4)	12 (7.7)
AEs leading to dose adjustment/interruption	6 (46.2)	66 (42.3)
AEs requiring additional therapy	10 (76.9)	112 (71.8)

^*^Among patients without CCyR at baseline (Japan *n* = 8; all patients *n* = 103)

*CCyR* complete cytogenetic response; *IS* international scale; *MMR* major molecular response; *MR*^*4*^ molecular response 4 (*BCR::ABL1*^IS^ ≤ 0.01%); *MR*^*4.5*^ molecular response 4.5 (*BCR::ABL1*^IS^ ≤ 0.0032%)

CCyR rate decreased from 50.0% at Week 24 to 37.5% at Week 48 and remained consistent thereafter until the 96-week cutoff date among Japanese patients treated with asciminib. This small decline in CCyR response rate is due to discontinuation of one patient prior to Week 48 assessment. However, the CCyR response rate at Week 96 in the Japanese patients was in line with the CCyR rates observed in the overall ASCEMBL population with asciminib (39.8%) [12]. One patient treated with asciminib showed deep molecular response (MR^4.5^, 7.7%) at Week 24; this patient had maintained response up to the last assessment at Week 96. The proportion of patients with MR^4.5^ in the overall population at Week 96 was 10.8% [12]. No patients harbored *BCR::ABL1* gene mutations at baseline or had developed newly emerged *BCR::ABL1* gene mutations by Week 96.

PROs such as MDASI-CML symptom severity scores suggest that the majority of patients treated with asciminib in the Japanese subgroup had a better health-related quality of life compared with the overall study population; however, changes did not reach a clinically meaningful threshold (using 1.5 points as a guide threshold of interpretation) [15].

Patients treated in the asciminib group had shown good adherence (with a median-dose intensity almost as high as the recommended asciminib total daily dose of 80 mg) and had lower discontinuation rates due to AEs compared to the bosutinib group (15.4% vs. 66.7%, respectively) despite the longer duration of exposure. Treatment with asciminib was ongoing in approximately 77% of the Japanese patients at Week 96, which is higher than the proportion of patients with ongoing treatment in the overall ASCEMBL population (53.5%) [12].

The safety profile of asciminib observed in this Japanese subgroup was comparable to that observed in the overall ASCEMBL study population at Week 96. The proportion of patients who reported at least one AE of any grade with asciminib in the Japanese subgroup (84.6%) was comparable to the rate reported in the overall population (91.0%). Neutropenia and thrombocytopenia were the most common AEs observed with asciminib in this Japanese subgroup as well as in the overall study population; whereas nasopharyngitis was more common in the Japanese population compared to the overall ASCEMBL population [12]. No deaths were reported during the 96-week follow-up period. The proportion of patients who reported any grade AEs at Week 96 (84.6%) did not increase substantially compared to Week 24 (76.9%) [11]. These results should be interpreted cautiously considering that the duration of exposure to asciminib was much longer to that of bosutinib by the data cutoff.

The burden of AEs continued to decrease in patients who continued treatment beyond 6 months in the asciminib-treated group. Most of the AEs observed in the asciminib arm presented within the first 6 months of treatment and rarely persisted beyond the initial time of presentation. Regardless of the longer duration of exposure, the safety and tolerability of asciminib in the Japanese patients remain comparable to that observed in the overall population. These findings suggest that asciminib is well tolerated over the longer duration of treatment, resulting in increased adherence which in turn helps more patients achieve deeper levels of response.

## Conclusions

Asciminib provided superior and durable improvements in terms of MMR and *BCR::ABL1*^IS^ ≤ 1% in Japanese patients with CML-CP previously treated with ≥ 2 TKIs. The clinical improvements observed at Week 24 with asciminib were sustained at Week 96 in Japanese patients. With the longer duration of asciminib exposure, the safety and tolerability continued to be better than those observed with bosutinib, with no new or worsening safety findings. Overall, the efficacy and safety results observed in the Japanese subgroup of patients are comparable with those from the ASCEMBL global study population. ASCEMBL 96-week results continue to support the use of asciminib as a new treatment option in Japanese patients with previously treated CML-CP.

## Data Availability

Novartis is committed to sharing with qualified external researchers access to patient-level data and supporting clinical documents from eligible studies. These requests are reviewed and approved by an independent review panel based on scientific merit. All data provided are anonymized to respect the privacy of patients who have participated in the trial consistent with applicable laws and regulations. This trial data availability is according to the criteria and process described on www.clinicalstudydatarequest.com.
